# Clinical‐functional brain connectivity signature predicts longitudinal symptom improvement after acupuncture treatment in patients with functional dyspepsia

**DOI:** 10.1002/hbm.26449

**Published:** 2023-08-16

**Authors:** Tao Yin, Yuzhu Qu, Yangke Mao, Pan Zhang, Peihong Ma, Zhaoxuan He, Ruirui Sun, Jin Lu, Yuan Chen, Shuai Yin, Qiyong Gong, Yong Tang, Fanrong Liang, Fang Zeng

**Affiliations:** ^1^ Acupuncture and Tuina School Chengdu University of Traditional Chinese Medicine Chengdu Sichuan China; ^2^ Acupuncture and Brain Science Research Center Chengdu University of Traditional Chinese Medicine Chengdu Sichuan China; ^3^ Key Laboratory of Sichuan Province for Acupuncture and Chronobiology Chengdu Sichuan China; ^4^ School of Acupuncture‐Moxibustion and Tuina Beijing University of Chinese Medicine Beijing China; ^5^ International Education College Chengdu University of Traditional Chinese Medicine Chengdu Sichuan China; ^6^ First Affiliated Hospital Henan University of Traditional Chinese Medicine Zhengzhou Henan China; ^7^ Departments of Radiology Huaxi Magnetic Resonance Research Center (HMRRC), West China Hospital of Sichuan University Chengdu Sichuan China

**Keywords:** acupuncture, functional connectivity, functional dyspepsia, machine learning, neuroimaging, personalized medicine

## Abstract

Whilst acupuncture has been shown to be an effective treatment for functional dyspepsia (FD), its efficacy varies significantly among patients. Knowing beforehand how each patient responds to acupuncture treatment will facilitate the ability to produce personalized prescriptions, therefore, improving acupuncture efficacy. The objective of this study was to construct the prediction model, based on the clinical‐neuroimaging signature, to forecast the individual symptom improvement of FD patients following a 4‐week acupuncture treatment and to identify the critical predictive features that could potentially serve as biomarkers for predicting the efficacy of acupuncture for FD. Clinical‐functional brain connectivity signatures were extracted from samples in the training‐test set (100 FD patients) and independent validation set (60 FD patients). Based on these signatures and support vector machine algorithms, prediction models were developed in the training test set, followed by model performance evaluation and predictive features extraction. Subsequently, the external robustness of the extracted predictive features in predicting acupuncture efficacy was evaluated by the independent validation set. The developed prediction models possessed an accuracy of 88% in predicting acupuncture responders, as well as an *R* ^2^ of 0.453 in forecasting symptom relief. Factors that contributed significantly to stronger responsiveness of patients to acupuncture therapy included higher resting‐state functional connectivity associated with the orbitofrontal gyrus, caudate, hippocampus, and anterior insula, as well as higher baseline scores of the Symptom Index of Dyspepsia and shorter durations of the condition. Furthermore, the robustness of these features in predicting the efficacy of acupuncture for FD was verified through various machine learning algorithms and independent samples and remained stable in univariate and multivariate analyses. These findings suggest that it is both feasible and reliable to predict the efficacy of acupuncture for FD based on the pre‐treatment clinical‐neuroimaging signature. The established prediction framework will promote the identification of suitable candidates for acupuncture treatment, thereby improving the efficacy and reducing the cost of acupuncture for FD.

## Graphical abstract

This study developed the machine learning models based on clinical‐functional brain connectivity signature to predict acupuncture effects for functional dyspepsia. The good prediction performance kept highly stable in different algorithms and across samples from different sites.

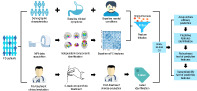

## 1 INTRODUCTION

Functional dyspepsia (FD) is a prevalent disorder of brain‐gut interaction that is characterized by recurrent symptoms such as postprandial bloating, early satiation, epigastric pain, epigastric burning, and, in some cases, psychiatric symptoms such as anxiety (Stanghellini et al., 2016). According to epidemiological surveys, FD affects approximately 6.9%–17.6% of the global population, with a higher prevalence rate among females, reaching up to 60% (Barberio et al., 2020). Despite not being life‐threatening, the symptoms of FD have a considerable adverse effect on the quality of life of patients (Ford et al., 2020) and impose a substantial financial burden on affected individuals (Brook et al., 2010).

Acupuncture has been proven to be an effective therapy for FD, significantly improving the clinical symptom and quality of life of patients, as well as alleviating their psychiatric symptoms (Ko et al., 2016; Ma et al., 2012; Yang et al., 2020). However, due to the complexity of individual conditions and the resulting differences in response to acupuncture stimulation, the efficacy of acupuncture treatment varies across different FD patients. This phenomenon raises two intriguing issues. First, is it possible to predict the individual responsiveness of FD patients to acupuncture treatment in advance? Second, which characteristics indicate that an FD patient is more likely to benefit from acupuncture treatment?

Previous studies have put a lot of work into forecasting acupuncture efficacy. The clinical phenotype (Green et al., 2018), the genotype (Genovese et al., 2021), and the neuroimaging endophenotype (Tu et al., 2019) have been found to be promising biomarkers for predicting individual responses to acupuncture treatment. A higher baseline pain level, for instance, was detected as a predictor of better symptom relief following acupuncture therapy in patients with chronic pain (Witt et al., 2019). Gender and disease subtypes (Yin, Zheng, et al., 2022), comorbidities, and symptom severity (Singh et al., 2022) were assigned as reliable indicators of how well FD patients responded to acupuncture and nonspecific pharmaceutical treatment, respectively. These findings provided preliminary validation of the feasibility of forecasting acupuncture efficacy based on baseline conditions and offered excellent examples for predicting the efficacy of acupuncture for FD.

The dysfunction of bidirectional communication between the central nervous system and the enteric nervous system is the central pathogenesis of FD (Tait & Sayuk, 2021). Evidence from neuroimaging studies has informed that disruption of the connectivity patterns of brain networks involved in central processing and modulation of FD‐related symptoms is an important central pathological characteristic of FD patients (Mayer et al., 2019). The resting‐state functional connectivity (rsFC) between the subcortical, default mode, sensorimotor, and cognitive control networks was demonstrated to be the potential biomarkers for discriminating FD patients from healthy individuals (Yin, Sun, et al., 2022). Moreover, several psychosocial factors, including anxiety and depression, were known to play significant roles in the susceptibility, development, and symptom flares of FD (Ly et al., 2015; van Oudenhove & Aziz, 2013). Therefore, we hypothesized that the disease‐related clinical and functional brain connectivity phenotypes had the potential to predict acupuncture efficacy for FD patients.

To test this hypothesis, we collected the demographic characteristics, self‐reported clinical and psychiatric symptom scores, and rsFC of the brain networks from 100 FD patients and then developed the prediction models with the multimodal clinical‐rsFC signature and machine learning algorithms, to forecast the longitudinal symptom improvement in FD patients following 4‐week acupuncture treatment and to identify the significant predictive features which could potentially serve as a biomarker for forecasting acupuncture efficacy for FD. Subsequently, 60 FD patients from a separate site were enrolled to further evaluate the robustness of these features in forecasting the efficacy of acupuncture. The proposed prediction models are anticipated to facilitate the implementation of precision medicine by enabling medical professionals to identify suitable candidates for acupuncture therapy and personalize treatment strategies based on individual characteristics, thereby enhancing the effects of acupuncture in treating FD while reducing healthcare expenses.

## 2 MATERIALS AND METHODS

### 2.1 Participants

Both a training‐test set and an independent validation set were enrolled. The training‐test set included 100 FD patients, who were retrospectively gathered from two randomized controlled neuroimaging trials (Sun et al., 2018; Yin et al., 2017). The independent validation set contained 60 FD patients, who were from an ongoing neuroimaging trial (Zhang et al., 2022).

The potential patients were diagnosed to be included by the gastroenterologists based on the Rome diagnostic criteria for FD as well as the results of the physical examinations and laboratory tests. Specifically, patients in the training‐test set were diagnosed according to the Rome III criteria (Tack et al., 2006), and patients in the independent validation set were diagnosed based on the Rome IV criteria (Stanghellini et al., 2016). The diagnostic standards for FD are congruent with the Rome III and IV criteria (Stanghellini et al., 2016). The inclusion and exclusion criteria were the same for the samples in the training‐test set and the independent validation set.

Patients who met the following inclusion criteria were included: (1) satisfied the Rome diagnostic criteria for FD; (2) were 18–40 years old; (3) were right‐handed; (4) were a college degree or above; (5) did not get regulated treatment for at least 2 weeks prior to enrollment; (6) had not participated in any clinical trial in the last 3 months; (7) had signed the informed consent.

Patients were excluded if they: (1) had other organic or metabolic diseases which may induce dyspepsia symptoms; (2) had other functional gastrointestinal diseases such as irritable bowel syndrome; (3) had chronic pain disorders or mental disorders; (4) had a history of gastrointestinal surgery, head trauma, brain lesions, or neurological disorders; (5) were pregnant or planning to get pregnant in the last 6 months; (6) had any contraindication of Magnetic Resonance Imaging (MRI) scan. The exclusion was determined based on the complaints of patients during the history taking.

### 2.2 Intervention

All FD patients underwent 20 sessions of acupuncture treatment over 4 weeks. The stimulated acupoints included the *Zusanli* (ST 36), *Weishu* (BL 21), and Zhongwan (CV 12). Depending on their group assignments, patients received acupuncture stimulation at one or two acupoints. Since these stimulated acupoints were disease‐related and proven to be effective (Dong et al., 2022; Kim et al., 2020; Teng et al., 2022), samples from different intervention groups were aggregated and utilized to develop and evaluate the prediction models. The details of the acupuncture interventions can be found in Supplementary 1.

### 2.3 Outcome measures

The severity of dyspepsia symptoms and dyspepsia‐specific quality of life of FD patients were evaluated using the Symptom Index of Dyspepsia (SID) (Ma et al., 2012) and Nepean Dyspepsia Symptom Index (NDSI) (Talley et al., 1999) questionnaires, as well as the Nepean Dyspepsia Life Quality Index (NDLQI) (Talley et al., 1999) questionnaire, respectively. In consideration of the interactions between dyspepsia symptoms and emotional disorders in FD patients, the emotional conditions of FD patients were assessed using the Self‐Rating Anxiety Scale (SAS), Self‐Rating Depression Scale (SDS), and Beck Depression Inventory (BDI). The clinical metrics were described with Mean ± Standard deviation (SD).

### 2.4 MRI data acquisition

All the FD patients underwent resting‐state MRI scanning at the baseline. The MRI data of the samples in the training‐test set were obtained using a 3.0 T Siemens magnetic resonance scanner at West China Hospital. The MRI data of the independent validation set were acquired with the same scanning parameters by another 3.0 T Siemens magnetic resonance scanner at Chengdu Fifth People's Hospital. The details of the scanning parameters are available in Supplementary 2.

### 2.5 MRI data analysis

#### 2.5.1 MRI data preprocessing and head motion control

The MRI data were preprocessed with the DPARSF4.1 toolbox (http://rfmri.org/DPARSF) at MATLAB 2017b. The preprocessing steps consisted of four stages: (1) discarding the first 10 timepoints to allow for signal stabilization; (2) slice‐timing and realigning; (3) normalizing the images to the MNI space and smoothing with a 4 mm Gaussian kernel of full‐width at half maximum; and (4) regressing out the covariates, including the six rigid‐body motion parameters, white matter, and cerebrospinal fluid signals, using the Component‐based Noise Correction Method (CompCor) (Ciric et al., 2017).

Given the impact of head motion on the reliability of rsFC analysis, we implemented strict head motion control criteria in this study. Specifically, in addition to the conventional exclusion criteria of head motion with translation >2 mm or rotation >2°, we further excluded subjects with mean framewise displacement >0.2, or a percentage of the framewise displacement exceeded 0.2 > 30%.

#### 2.5.2 Functional brain network construction

To construct the individual‐level functional brain network, we performed the independent component analysis (ICA)‐based network components definition analysis in the training‐test set, which has been shown to provide a more accurate reflection of the connectivity structures of the brain (Calhoun & de Lacy, 2017). Eventually, a total of 35 independent components (ICs) belonging to the subcortical network (SCN, including 6 ICs), sensorimotor network (SMN, including 10 ICs), cognitive control network (CCN, including 10 ICs), and default mode network (DMN, including 9 ICs) were identified, as these ICs have been shown to be potentially valuable in discriminating FD patients and interpreting the effects of acupuncture for FD in our previous studies (Yin, He, et al., 2022; Yin, Sun, et al., 2022). Thereafter, the time series of the identified ICs were extracted and the 35 × 35 functional brain networks were constructed for each patient in the training‐test set. Taking the ICs obtained in the above analysis as the seeds, the time series of patients from the independent validation set was extracted, and the functional brain networks were subsequently constructed for each of these patients.

Additional details on ICA and functional brain network construction can be accessed in our recent study^16^ and Supplementary 3. Spatial maps and detailed information on these selected ICs are shown in Supplementary 4.

### 2.6 Prediction models development and evaluation

#### 2.6.1 Feature

The edges (rsFC of two ICs) of the functional brain networks were considered as the rsFC features. Additionally, the demographic characteristics (including gender, age, weight, and height), the baseline clinical metrics (including SID, NDSI, and NDLQI), and the baseline emotional conditions (including SAS, SDS, and BDI) were taken as the clinical features.

#### 2.6.2 Label

The primary purpose of the prediction model was to predict the responsiveness of FD patients to acupuncture treatment. Therefore, patients were classified into two categories based on the improvement in the SID score (Ma et al., 2012). Those who exhibited an improvement in SID score of at least two points after treatment were designated as acupuncture responders and were assigned a label of “1”. Non‐responders, who demonstrated an improvement in the SID score of lower than two points, were assigned a label of “–1”.

In addition, the prediction model was also utilized to predict the symptom improvement in each patient after acupuncture treatment. In this case, the improvement in the SID score served as the labels for the prediction models.

#### 2.6.3 Algorithm

The support vector machine (SVM) algorithms were utilized to develop the prediction models. Specifically, the support vector classification (SVC) algorithm was utilized to predict acupuncture responders, while the support vector regression (SVR) algorithm was applied to predict SID improvement. All the SVM analyses were performed using the LIBSVM toolbox (https://www.csie.ntu.edu.tw) in MATLAB 2017b, with the linear kernel and default regularization parameter (*c =* 1), as recommended (Dadi et al., 2019).

#### 2.6.4 SVC model development and assessment

Given the potential “Curse of dimensionality” introduced by high‐dimensional features, it is necessary to perform feature selection prior to establishing a prediction model. This study utilized the “*Filtering*” feature selection approach (Guyon & Elisseeff, 2006) based on the *Spearman* correlation coefficient between the label and each feature in the training set samples, to reduce the uninformative features. Thereafter, the prediction models were developed based on the retained features of the training set samples, and were used to predict the labels of samples in the test set.

Since different thresholds of feature selection led to a varying number of retained features in the filtering process, the performance of prediction models constructed based on these features also varies. Therefore, a total of 100 thresholds ranging from 0.005 to 0.5 with a step length of 0.005 were traversed in the feature selection process to find the optimal filtering threshold.

The model evaluation was placed within a 10‐fold cross‐validation (10‐fold CV) framework to avoid circularity bias. The accuracy, sensitivity, specificity, and area under the receiver operating characteristic curve (AUC) were used to evaluate the performance of the SVC model. The statistical significance of the prediction results was assessed with the permutation test (repetition time = 1000). Specifically, labels of the data (acupuncture responsiveness) were first randomly permuted before model training. Cross‐validation was then performed on the permuted dataset, and the procedure was repeated 1000 times. If the model trained on real data labels had a better prediction performance exceeding the 95% of the models trained on random data labels, the prediction model was considered to be significant.

Considering the sample distribution in the 10‐fold CV was random, the above analysis procedure was iterated 100 times. The performance of the model was finally determined by calculating the mean accuracy, sensitivity, specificity, and AUC over the 100 iterations.

#### 2.6.5 Predictive features identification

The features that were preserved as support vectors in all 10‐fold CV were designated as the consensus features. The consensus features that occurred in all 100 iterations was identified as the critical predictive features. The weights of the predictive features reflect the magnitude of their contributions to determining the support vector classification hyperplane. The extraction and comparison of the predictive features and their corresponding weights enable us to quantify the relative contribution of each feature to the predicted outcomes. Analogous to the assessment of model performance, the weight of each predictive feature was ultimately determined by the absolute value of the mean over the 100 iterations.

To determine the contributions of every network to the prediction performance, a network‐scale weight calculation was performed for the four networks (SCN, SMN, CCN, and DMN). Specifically, the weight of the networks was calculated by summing the weights of all predictive features contained within the network.

To compare the group‐level differences in the clinical‐rsFC signature between acupuncture responders and nonresponders, the two‐sample *t*‐tests were performed on these predictive features. Due to the differences in occurrence rates between males and females, gender was taken as a covariate. The significance threshold was set to two‐tailed *p* < .05, with False Discovery Rate (FDR) correction.

#### 2.6.6 SVR model development and assessment

Based on the identified predictive features, the SVR prediction model was developed with the 10‐fold CV to predict the improvement of SID in each patient. The performance of the model was assessed using the coefficient of determination (*R* ^2^) and mean squared error (MSE). To assess the statistical significance of *R* ^2^ and MSE, the permutation tests were performed with a permutation time of 1000. To mitigate the random errors inherent in cross‐validation, the prediction analysis was repeated 100 times.

### 2.7 Robustness of predictive features in different machine learning algorithms

To test the robustness of the extracted predictive features in predicting acupuncture efficacy, we established six classification models and five regression models with these extracted predictive features and then evaluated the performance of these models. Specifically, classification models were constructed with the decision tree, linear discrimination, logistic regression, k‐nearest neighbor, gaussian SVM, and bagged tree, to predict the responsiveness of FD patients. Regression models were constructed with linear regression, decision tree, Gaussian SVM, bagged tree, and Gaussian process regression, to predict the SID improvement of patients. These above analyses were all performed with the built‐in toolbox of Matlab 2017b under the 10‐fold CV framework.

### 2.8 Generalizability of predictive features in the independent validation set

To further test the generalizability of the predictive features, we developed the prediction models based on the extracted predictive features in the independent validation set. Consistent with the analysis in the training test set, the performance of the SVC model was evaluated by accuracy, sensitivity, specificity, and AUC, and the performance of the SVR model was assessed with *R* ^2^ and MSE at the framework of 10‐fold CV.

**FIGURE 1:**
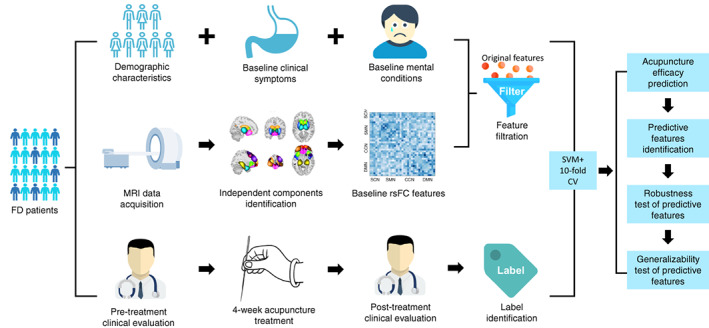
Flow chart of the prediction analysis.

The flow chart of the prediction analysis is illustrated in Figure 1.

## 3 RESULTS

### 3.1 Demographic and clinical characteristics

Eleven patients from the training‐test set and two patients from the independent validation set were excluded because of the excessive head motion. All the remaining patients finished the 4‐week course of acupuncture treatment. Therefore, 89 FD patients were finally included in prediction model construction and predictive features identification. Fifty‐eight patients were taken as independent samples to verify the generalizability of the predictive features. The demographic and clinical characteristics of samples in the training‐test set and independent validation set are shown in Table 1.

**TABLE 1:** The demographic and clinical characteristics of samples in training‐test set and independent validation set.

	Training‐test set (mean ± SD)	Independent validation set (mean ± SD)
Gender (Male/Female)	17/72	24/34
Age (Year)	22.08 ± 2.16	21.95 ± 2.49
Height (cm)	160.90 ± 6.85	165.12 ± 9.09
Weight (kg)	51.27 ± 7.55	56.98 ± 10.61
Duration (Month)	42.49 ± 33.16	33.81 ± 23.8
SID	3.75 ± 1.46	3.93 ± 1.63
NDSI	44.70 ± 14.23	46.07 ± 18.9
NDLQI	77.04 ± 9.4	77.31 ± 10.79
SAS	42.25 ± 8.1	42.79 ± 9.64
SDS	44.06 ± 10	45.38 ± 11.2
BDI	7.88 ± 5.02	7.05 ± 5.14

In accordance with the pre‐established criteria for label identification, 51 patients from the training‐test sample and 35 patients from the independent validation sample were categorized as acupuncture responders. Conversely, 38 patients from the training‐test sample and 23 patients from the independent validation sample were classified as nonresponders.

### 3.2 The optimal threshold for feature selection

**FIGURE 2:**
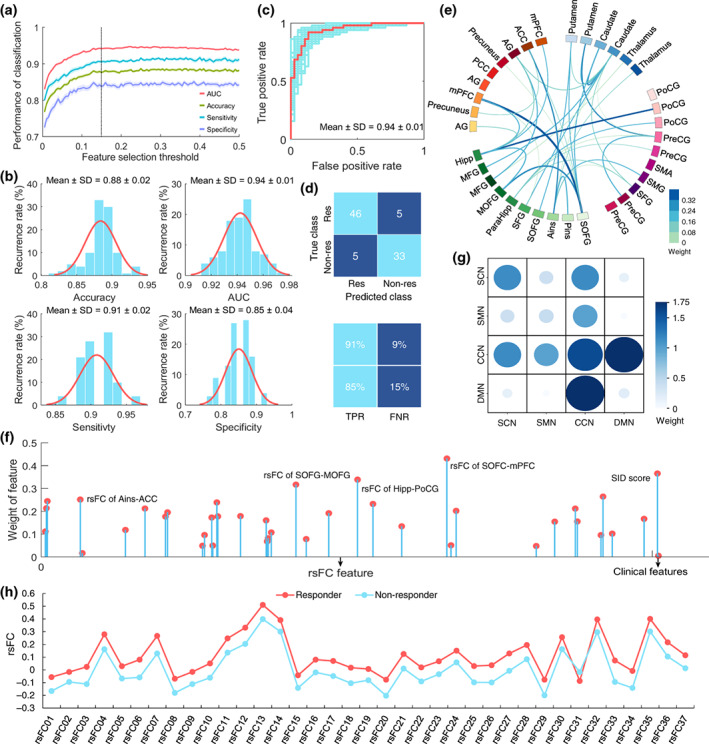
Performance of the SVC model in training‐test set and the critical predictive features. (a) Performance of the models at various feature selection thresholds. (b) Performance of the models across the 100 iterations at the optimal feature selection threshold. (c) Receiver Operating Characteristic curves of the prediction models across the 100 iterations. (d) The average confusion matrix of the prediction results crosses the 100 iterations. (e) The 37 rsFC predictive features and their corresponding weights. The thicker line in the Circos diagram indicates the greater weight of the rsFC feature. (f) The weights of the 39 predictive features. (g) The weights of features on the network scale. A bigger dot in the matrix indicates a greater weight of the network. (h) The comparison of the 37 rsFC features between acupuncture responders and nonresponders. Except for the rsFC31, responders had stronger connectivity than nonresponders (*p*_*FDR*_ < .05). AG, angular gyrus; Ains, anterior insula; FNR, false negative rate; Hipp, hippocampus; MOFG, middle orbitofrontal cortex; Non‐res, nonresponders; ParaHipp, parahippocampus; PCC, posterior cingulate cortex; Pins, posterior insula; PMFG, middle frontal cortex; PoCG, postcentral gyrus; PreCG, precentral gyrus; Res, responders; SFG, superior frontal cortex; SMA, supplementary motor area; SMG, supramarginal gyrus; SOFG, superior orbitofrontal cortex; TPR, true positive rate.

The SVC prediction models were established by varying the feature selection thresholds from 0.005 to 0.5. As shown in Figure 2a, the models showed consistent performance when the feature selection threshold reached 0.15. As a result, the feature selection threshold was assigned to α = 0.15, and the features retained under this threshold were applied to construct the prediction models.

### 3.3 Performance of the SVC model

The developed SVC model yielded an accuracy of 0.88 ± 0.02, a sensitivity of 0.91 ± 0.02, a specificity of 0.85 ± 0.04, and an AUC of 0.94 ± 0.01 in forecasting acupuncture responders and nonresponders over the 100 iterations (Figure 2b–d). The significance of the accuracy and AUC scores were confirmed by the permutation tests (*p*_accuracy_ < .001, *p*_AUC_ < .001).

### 3.4 Predictive features and weights

A total of 39 features (including 37 rsFC features, baseline SID, and duration of disease) were identified as the predictive features for the accurate prediction of acupuncture responsiveness of FD patients. Among these 37 rsFC features, 9 features were associated with caudate, 7 with superior orbitofrontal gyrus (SOFC), and 6 with anterior insula and hippocampus, respectively (Figure 2e).

The top five predictive features with the highest weights were the rsFC of SOFC‐medial prefrontal cortex (mPFC) (weight = 0.431), baseline SID score (weight = 0.365), rsFC of hippocampus‐postcentral gyrus (weight = 0.339), rsFC of SOFC‐middle orbitofrontal cortex (weight = 0.317), and rsFC of anterior insula‐anterior cingulate cortex (ACC) (weight = 0.264) (Figure 2f). Detailed information on these 39 predictive features is available in Supplementary 5.

The result of the network‐scale weight calculation demonstrated that the rsFC within the CCN and between the CCN and the DMN, SCN, and SMN made the biggest contributions to the prediction. (Figure 2g).

Two sample *t*‐tests showed significant differences in 36 rsFC features (except rsFC31), baseline SID, and duration of disease between acupuncture responders and nonresponders (Supplementary 6). In particular, the responders exhibited higher connectivity in these 36 rsFC features (Figure 2h), higher baseline SID score, and shorter disease duration compared to the nonresponders (*p*_*FDR*_ < .05).

### 3.5 Performance of the SVR model

**FIGURE 3:**
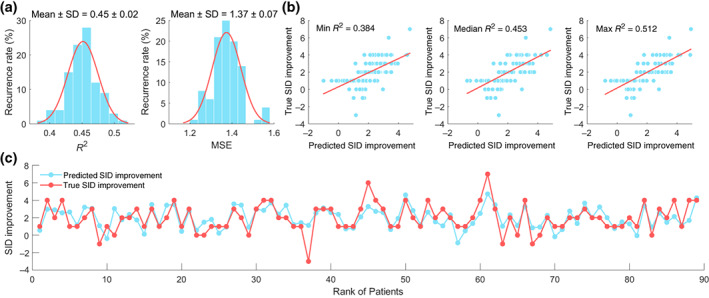
Performance of the SVR model in training‐test set for predicting SID improvement (a) the *R* ^2^ and MSE scores over the 100 iterations. (b) The correlation scatters of the true and the predicted SID improvement under the minimal, the median, and the maximum *R* ^2^. (c) The correspondence between the true and the mean of predicted SID improvement over the 100 iterations.

Utilizing the aforementioned 39 predictive features, an SVR model was developed for the prediction of SID improvement in FD patients. In the 100 iterations, the SVR model achieved an *R* ^2^ of 0.45 ± 0.02 and an MSE of 1.37 ± 0.07, as depicted in Figure 3a. Significance of both the *R* ^2^ and MSE scores was confirmed through permutation tests (*p*_R_^2^ < .001, *p*_MSE_ < .001). The correlation scatter plots, depicting the relationship between the true SID improvement and the predicted SID improvement under the minimal, median, and maximum *R* ^2^ scores across the 100 iterations, are illustrated in Figure 3b. The correspondence between the true SID improvement and the mean predicted SID improvement over the 100 iterations was displayed in Figure 3c.

### 3.6 Robustness of the predictive features in different machine learning algorithms

**FIGURE 4:**
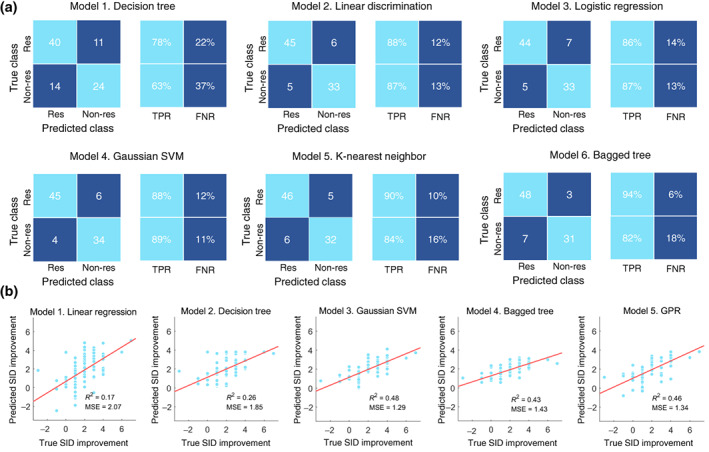
Robustness of predictive features in different machine learning algorithms. (a) Performance of the six classification models constructed with the identified predictive features. (b) Performance of the five regression models constructed with the identified predictive features. FNR, false negative rate; GPR, Gaussian process regression; Non‐res, nonresponders; Res, responders; TPR, True positive rate.

We further test the robustness of these 39 predictive features in predicting the efficacy of acupuncture for FD with 11 machine‐learning algorithms. The sensitivity and specificity of the six classification algorithms were 78%–94% and 63%–89%, respectively (Figure 4a). The *R* ^2^ ranged from 0.17 to 0.48, and MSE ranged from 2.07 to 1.29 in the five regression models (Figure 4b).

### 3.7 Generalizability of the predictive features in an independent validation set

**FIGURE 5:**
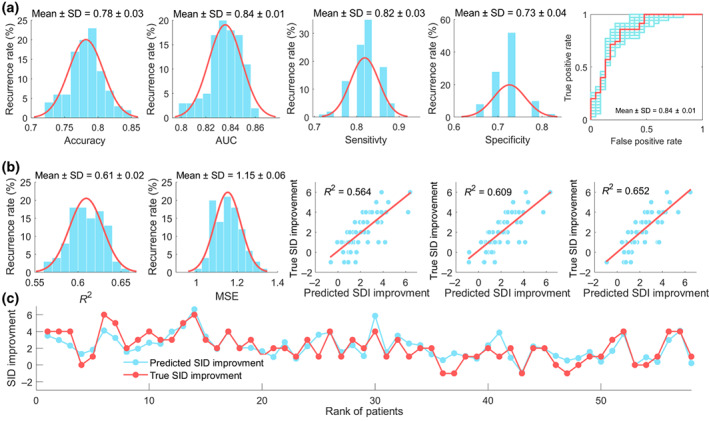
Generalizability of the predictive features in the independent validation set. (a) Performance of the predictive features in forecasting acupuncture responsiveness. (b) Performance of the predictive features in predicting SID improvement. (c) The correspondences between the true and the mean of predicted SID improvement over the 100 iterations.

In the independent validation set, the SVC model got an accuracy of 0.78 ± 0.03, a sensitivity of 0.82 ± 0.03, a specificity of 0.73 ± 0.04, and an AUC of 0.84 ± 0.01 in predicting acupuncture responders. The SVR model achieved an *R* ^2^ score of 0.61 ± 0.02 and an MSE score of 1.15 ± 0.06 in predicting SID improvement. The permutation tests conducted for each of the aforementioned prediction scores were all statistically significant (*p* values for accuracy, AUC, *R* ^2^, and MSE were < .001) (Figure 5).

## 4 DISCUSSION

This study represented the initial endeavor to forecast the efficacy of acupuncture based on the multimodal clinical‐neuroimaging signature. The findings suggested that the combination of clinical and rsFC features effectively predicted the longitudinal symptom improvement in FD patients following acupuncture treatment. Moreover, the robustness and generalizability of these features in predicting acupuncture efficacy for FD patients were validated by multiple machine learning algorithms and samples from independent sites and remained stable in both univariate and multivariate analyses.

The application of machine learning proved a promising approach to characterize the individual condition of patients and predict their responsiveness to treatment (Hessulf et al., 2023; Zhang et al., 2021). The most important step in machine learning‐based prediction analysis is the identification of appropriate features (Akinola et al., 2022). Compared to the prediction models developed with a single type of feature, the models constructed based on multimodal features are thought to provide a more thorough representation of patients and thus generally have better performance (Bhatt et al., 2022; Koutsouleris et al., 2021). For example, compared to the single neuroimaging features, the integrated clinical‐brain signature more accurately predicted symptom relief in patients with irritable bowel syndrome following the untargeted intervention (Bhatt et al., 2022). Similarly, our recent study constructed a prediction model based on clinical features and obtained an accuracy of 77.3% in predicting the responsiveness of FD patients to acupuncture treatment (Yin, Zheng, et al., 2022). While in the current study, the prediction accuracy was significantly improved to 88% by integrating the clinical data and rsFC features. These findings supported our hypothesis that applying disease‐related clinical and functional brain connectivity phenotypes to predict acupuncture efficacy for FD and provided new insight into feature selection in the following prediction analysis of acupuncture efficacy. Specifically, selecting multi‐dimensional features rather than a single mode of features to characterize patients' condition more appropriately and therefore improving the performance of the prediction analysis.

The baseline SID score was detected as a critical feature for the prediction of acupuncture efficacy. These FD patients with higher baseline SID scores got better symptom relief after acupuncture treatment. These results suggested that the self‐reported baseline symptoms determined to some extent the longitudinal symptom improvement after acupuncture treatment in FD patients. Similarly, the substantial correlation between baseline illness severity and treatment outcomes has also been noted in several recent predictive analyses on functional gastrointestinal diseases (Ballou et al., 2018; Singh et al., 2022; Wang et al., 2019). A prospective study demonstrated that the higher baseline dyspepsia severity was positively associated with greater symptom improvement in FD patients after treatment (Singh et al., 2022), while a retrospective study showed that the higher baseline constipation symptom score signaled a longer duration of acupuncture efficacy in patients with functional constipation (Wang et al., 2019). Together, these findings suggested that the more severe self‐reported clinical symptoms at baseline tend to predict more remarkable symptom improvements after treatment in patients with functional gastrointestinal diseases. One possible explanation for these interesting phenomena was that patients with more severe symptoms were more willing and compliant to take part in acupuncture trials. Although it is debatable whether or not treatment anticipation affects acupuncture effectiveness (Yang et al., 2022), a positive attitude toward participation in the trial would enhance the treatment response of patients.

The rsFC within the CCN and between CCN and the other three sub‐networks made large contributions to the prediction results. Patients with higher connectivity of these above rsFC features had better symptom relief after acupuncture treatment. These findings suggested that the stronger multi‐networks collaboration centered on the CCN portended greater benefits for FD patients from acupuncture treatment. The CCN is the processing center of the cognitive and executive functions, responsible for cognition, planning, attention, and appropriate behaviors to achieve a specific goal (Breukelaar et al., 2017); the SCN and SMN are involved in visceral sensory signaling, encoding, and processing (Mayer et al., 2015); while the DMN in charge of self‐perception and negative rumination of noxious memory (Yeshurun et al., 2021). The recurrence of the gastrointestinal symptoms such as abdominal pain and bloating, as well as the extra‐gastrointestinal symptoms such as anxiety and depression, on the one hand, induces sensitization of the SCN, SMN, and DMN associated with visceral sensory processing and unpleasant memory rumination, and on the other hand, activates the CCN and motivates patients to make adaptive decisions, such as seeking the therapy proactively and maintaining high compliance during treatment to achieve better outcomes. This explained why the rsFC between the CCN and the other three networks could predict the efficacy of acupuncture for FD patients.

Taking the specific features into account, the SOFC, caudate, hippocampus, anterior insula, ACC, and mPFC‐related rsFC features manifested higher weights. The anterior insula, mPFC, ACC, and hippocampus are extensively involved in visceral sensory signal processing and feedback, appraisal and response to the affective aspect of visceral sensation, and rumination of the negative memories (e.g., pain, unpleasantness, anxiety, etc.) (Kano et al., 2018). As shown in the previous studies, the functional activity and connectivity patterns of the hippocampus, anterior insula, and mPFC were correlated with the disease severity in FD patients (Beckers et al., 2022; Zeng et al., 2011), and acupuncture treatment could significantly modulate the functional activity and connectivity patterns of the mPFC, ACC, and anterior insula of FD patients (Teng et al., 2022; Zeng et al., 2012). It suggested that these regions were the critical pathologic aspects of the disease as well as the targets for acupuncture treatment, and therefore could be severed as the predictive features for the prediction of acupuncture efficacy in FD patients. The orbitofrontal cortex and caudate are the core regions of the reward pathway and are involved in reward representation, anticipation, and reward‐specific decision‐making and behaviors (Murray & Rudebeck, 2018). The incentive and motive for FD patients to take part in the acupuncture trials, or alternately, the reward expectation, is to achieve considerable symptom reduction. The adaptive decisions made in response to this reward expectation, such as willingness to participate, higher expectation of efficacy, and better compliance during treatment, are all positive influences for better acupuncture outcomes (Ho et al., 2021). Therefore, patients with higher rsFC to caudate and orbitofrontal cortex achieved better treatment outcomes.

### 4.1 Limitations

There were several limitations should be considered. First, even though a sizable sample size (89 in the training‐test set and 58 in the independent validation set) was enrolled to develop and evaluate the prediction models crossly and independently, it is still necessary to validate the generalization of models with a big sample size third‐party data. Second, the cross‐validation approach used in model assessment prevented the creation of a fixed prediction model that could be reused because the primary purpose was to determine whether the clinical‐rsFC signature could predict acupuncture efficacy for FD and to identify the significant predictive features. After revalidating the generalization of predictive features with the third‐party data, we will develop a transplantable prediction model and form an interfaced toolbox for open use. Third, significantly fewer men than women were incorporated in model development. Therefore, the performance of these prediction models may be biased when applied in male FD patients. Fourth, although the prediction accuracy of 88% is gratifying, we will keep working to improve the prediction model's performance in the following research. Given that FD is a disorder of brain‐gut interaction, it is worthwhile to incorporate additional possible variables, such as gut microbiota and gastrointestinal immunity, into the existing multimodal signature to strengthen the future predictive performance of models.

## 5 CONCLUSION

This study verified the feasibility and reliability of predicting the efficacy of acupuncture for FD based on the pre‐treatment clinical‐rsFC signature for the first time. The current findings supported the administration of proactive acupuncture treatment to FD patients who have self‐reported symptoms that are more severe, last for a shorter time, and have higher rsFC within the CCN and between CCN and the DMN, SCN, and SMN. This may have implications for treatment in the era of precision medicine and further encourage the precision and personalization of acupuncture treatment, thereby improving its efficacy and cutting costs.

## AUTHOR CONTRIBUTIONS

**Tao Yin:** Methodology, conceptualization, software, writing–original draft. **Yuzhu Qu:** Formal analysis, investigation. **Yangke Mao:** Investigation, validation. **Pan Zhang:** Visualization, investigation. **Peihong Ma:** Resources. **Zhaoxuan He:** Validation. **Ruirui Sun:** Investigation. **Jin Lu:** Investigation. **Yuan Chen:** Resources. **Shuai Yin:** Investigation. **Qiyong Gong:** Data Curation. **Yong Tang:** Writing–review & editing. Fanrong Liang: Supervision. **Fang Zeng:** Conceptualization, writing–review & editing, project administration, funding acquisition.

## FUNDING INFORMATION

The current study was supported by the National Science Fund for Distinguished Young Scholars (grant number 82225050), the National Natural Science Foundation of China (grant numbers 81973960, 81622052, and 81473602), and the Sichuan Science and Technology Program (grant numbers 15QNJJ0008 and 2019JDTD0011).

## CONFLICT OF INTEREST STATEMENT

The authors declared that they have no competing interests.

## Supporting information


**Data S1:** Supporting Information.

## Data Availability

The main data supporting our findings can be found within the manuscript and supplementary files. Access to the raw data can be provided upon request.
