# Supplementary material for: Clinical‐functional brain connectivity signature predicts longitudinal symptom improvement after acupuncture treatment in patients with functional dyspepsia
Source: Hum Brain Mapp. 2023 Aug 16;44(16):5416–28. doi: 10.1002/hbm.26449 (PMC10543106; doi:10.1002/hbm.26449)
Supplement: Supplementary file 1 — Data S1: Supporting Information. [file HBM-44-5416-s001.docx]

**Supplementary files**

**Clinical-functional brain connectivity signature predicts longitudinal symptom improvement after acupuncture treatment in patients with functional dyspepsia**

**Authors:**

Tao Yin, Yuzhu Qu, Yangke Mao, Pan Zhang, Peihong Ma, Zhaoxuan He, Ruirui Sun, Jin Lu, Yuan Chen, Shuai Yin, Qiyong Gong, Yong Tang, Fanrong Liang, Fang Zeng

**Corresponding author:**

**Fang Zeng**, Acupuncture and Tuina School, Acupuncture and Brain Science Research Center, Chengdu University of Traditional Chinese Medicine; E-mail address: [zengfang@cdutcm.edu.cn](mailto:zengfang@cdutcm.edu.cn).

**Supplementary 1**

**Details of acupuncture prescriptions and manipulation**

All patients received 20 sessions of manual acupuncture in the 4-week treatment phase (once a day for 5 days continuously, with 2 days intervals). The acupoints prescriptions included a. *Weishu* (BL 21), b. *Zhongwan* (CV 12), c. *Zusanli* (ST 36), d. *Zhongwan* (CV 12) + *Weishu* (BL 21), e. *Zhongwan* (CV 12) + *Zusanli* (ST 36) **(Figure S1)**. Patients in the training-test set received acupuncture treatment with one of the above prescriptions according to the group assignments. Patients in the independent validation set were treated with the prescription of *Zhongwan* (CV 12) + *Zusanli* (ST 36). Since *Weishu* and *Zusanli* are bilateral acupoints, the left and right sides were used alternately in each session of acupuncture treatment.

Acupuncture manipulation was performed by licensed acupuncturists with clinical experience over 3 years. After skin disinfection, the disposable sterile filiform needles (0.25 × 40 mm, *Huatuo* Medical Instrument Co., Ltd., China) were perpendicularly inserted into acupoints at a depth of 20-30 mm, followed by the bi-directionally twisting needles at 90°-180°, lifting and thrusting needles with the amplitude of 3-5 mm for 1-1.5 Hz to induce *deqi* sensation. After *deqi* sensation was attained, needles were retained at the acupoints for 30 min. During the 30 min, the above procedures were manipulated for 10-15 s every 10 min to maintain the *deqi* sensation.


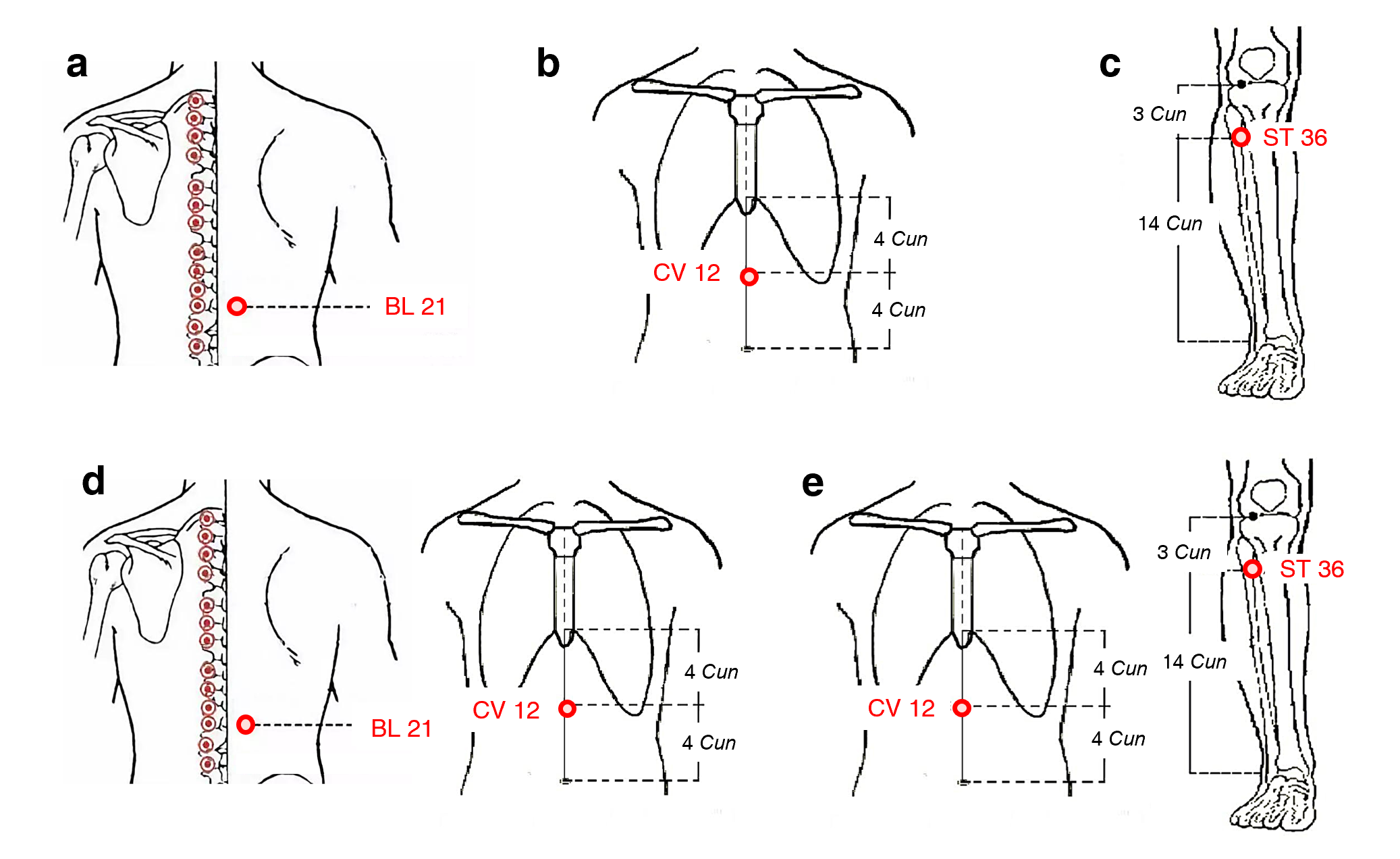


**Figure S1.** The location of acupoints in each prescription

**Supplementary 2**

**Details of MRI data acquisition**

Subjects were required to keep their heads still and stay awake during the scan, with eyes closed and ears plugged. Parameters of the high-resolution 3-dimensional T1-weighted imaging were as follows: repetition time = 1900 ms, echo time = 2.26 ms, slice thickness = 1 mm, slice number = 156, matrix size = 128×128, and field of view = 256×256 mm^2^. The blood oxygen level-dependent MRI data were acquired with the echo-planar imaging: repetition time = 2000 ms, echo time = 30 ms, flip angle = 90°, slice number = 30, matrix size = 64×64, field of view = 240×240 mm^2^, slice thickness = 5 mm, time point = 180 (training-test set) or 240 (independent validation set).

**Supplementary 3**

**Details of ICA and functional brain network construction**

The spatial ICA was performed with the GIFT v3.01 toolbox (http://icatb.sourceforge.net). Firstly, the Infomax algorithm(Bell and Sejnowski, 1995) was applied to decompose the fMRI data of all participants. Then, the two-step component selection approach(Allen et al., 2014) was utilized to estimate ICs. Namely, for the subject-wise data reduction, a total of 120 principal components were reserved, while for the group-level data reduction, the reserved components were concatenated and then were decomposed into 100 ICs. Finally, the subject-wise spatial maps and corresponding time courses of each IC were back-projected from the group spatial maps using GICA back reconstruction algorithm(Calhoun et al., 2001). The reliability and stability of the Infomax algorithm were evaluated with ICASSO(Himberg et al., 2004) (iterations = 20). The ICs with average internal similarity less than 0.8 were excluded(Fiorenzato et al., 2019).

ICs selection was processed after excluding ICs with low internal similarity. Firstly, the group-level one-sample *t*-test was conducted on the retained ICs to map their spatial localization. And then, referencing the selection method recommended by Allen et al.(Allen et al., 2014), ICs fulfilled the following criteria were reserved: 1) absolute *t*-value of the peak coordinate was greater than 10, and 2) overlapped with the known grey matter regions, and 3) time courses of ICs dominated by low-frequency signals and presented with a high dynamic range. Finally, a sorting function of spatial regression was used to estimate the similarity between the IC’s spatial image and the default RSN template in GIFT. According to the anatomical and presumed functional properties, the reserved ICs were sorted into the functional brain networks.

Afterward, time courses of the reserved ICs were post-processed with the following steps to remove the physiological and scanning noises: 1) detrended linear, quadratic, and cubic trends; 2) despiked the detected outliers with 3dDespike algorithm; 3) filtered with a high-frequency cutoff of 0.15 Hz; and 4) regressed out the six rigid-body motion parameters. After data postprocessing, the *Pearson’s* correlation analysis between each IC pair was conducted to estimate the functional brain network for subjects, following by *Fisher's* *z* transform for these networks to maintain normal distribution.

**Reference**

Allen, E.A., Damaraju, E., Plis, S.M., Erhardt, E.B., Eichele, T., Calhoun, V.D., 2014. Tracking whole-brain connectivity dynamics in the resting state. Cereb Cortex 24, 663-676.

Bell, A.J., Sejnowski, T.J., 1995. An information-maximization approach to blind separation and blind deconvolution. Neural Comput 7, 1129-1159.

Calhoun, V.D., Adali, T., Pearlson, G.D., Pekar, J.J., 2001. A method for making group inferences from functional MRI data using independent component analysis. Hum Brain Mapp 14, 140-151.

Fiorenzato, E., Strafella, A.P., Kim, J., Schifano, R., Weis, L., Antonini, A., Biundo, R., 2019. Dynamic functional connectivity changes associated with dementia in Parkinson's disease. Brain 142, 2860-2872.

Himberg, J., Hyvärinen, A., Esposito, F., 2004. Validating the independent components of neuroimaging time series via clustering and visualization. Neuroimage 22, 1214-1222.

**Supplementary 4**

**The details of these 35 identified ICs**

A total of 35 ICs belonging to the subcortical network, sensorimotor network, cognitive control network, and default mode network were identified and utilized to construct the functional brain network. The spatial maps and detailed information of these 35 identified ICs are illustrated in **Figure S2** and **Table S1.**


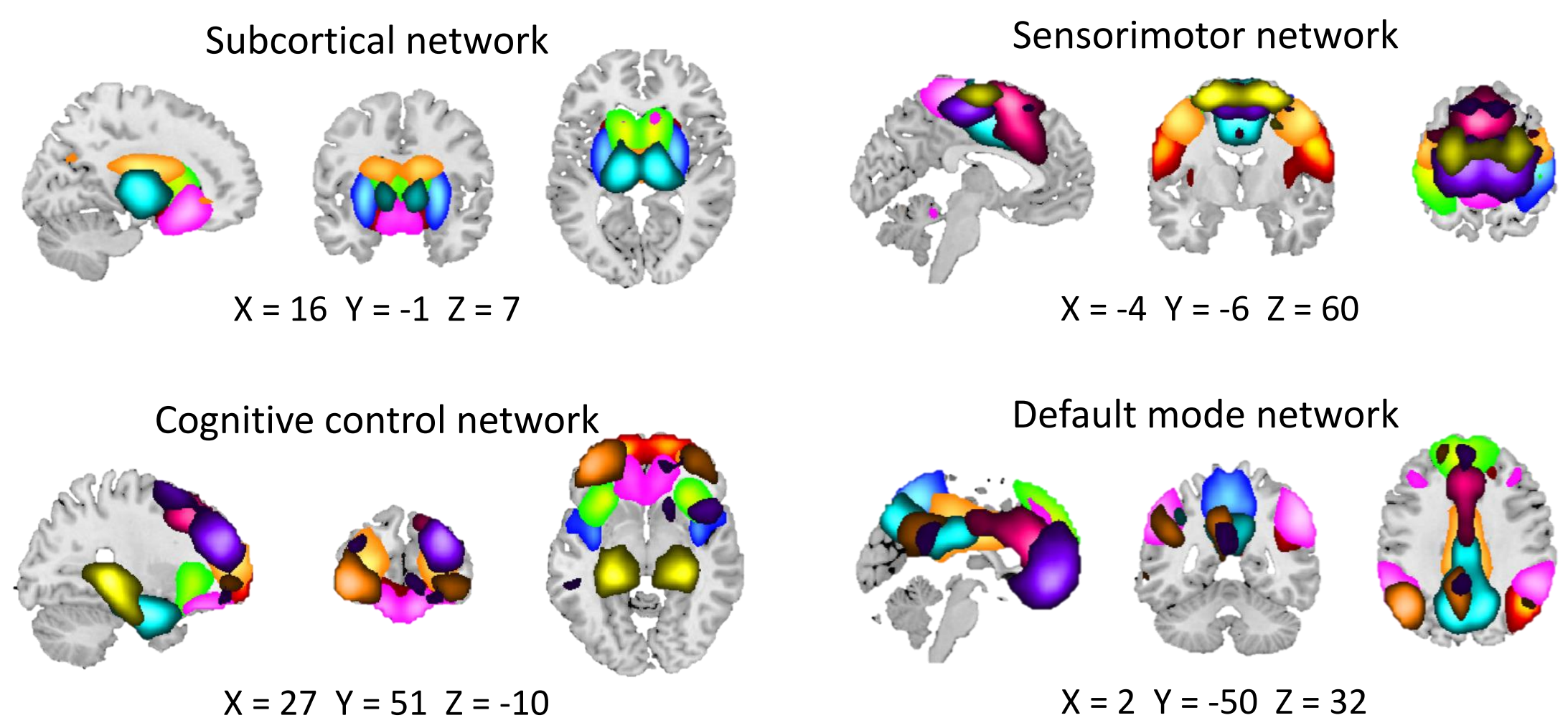


**Figure S2.** The spatial maps of the 35 identified ICs

**Table S1.** The detailed information of the 35 identified ICs

| **Independent components (ICs)** | | **Internal similarity** | **Peak \|*t*\|** | | **MNI Coordinate (X, Y, Z)** | | |
| --- | --- | --- | --- | --- | --- | --- | --- |
| **Subcortical Networks (SCN)** | | | | | | | |
| IC1 | Putamen | 0.978 | 55.76 | -24 | | 9 | -6 |
| IC8 | Putamen | 0.977 | 48.65 | -27 | | 0 | -3 |
| IC23 | Caudate | 0.974 | 51.16 | 12 | | 6 | 9 |
| IC33 | Caudate | 0.969 | 40.15 | 9 | | 18 | -9 |
| IC69 | Thalamus | 0.877 | 31.09 | -15 | | -15 | 18 |
| IC87 | Thalamus | 0.924 | 45.93 | -12 | | -18 | 3 |
| **Sensorimotor Networks (SMN)** | | | | | | | |
| IC2 | Postcentral gyrus | 0.982 | 53.89 | 57 | | -6 | 30 |
| IC3 | Postcentral gyrus | 0.982 | 45.12 | 39 | | -21 | 63 |
| IC5 | Postcentral gyrus | 0.982 | 46.18 | -39 | | -30 | 60 |
| IC6 | Precentral gyrus | 0.980 | 49.40 | 0 | | -27 | 66 |
| IC21 | Precentral gyrus | 0.975 | 36.55 | 48 | | -6 | 51 |
| IC35 | Supplementary motor area | 0.970 | 44.18 | 3 | | -9 | 54 |
| IC49 | Supramarginal gyrus | 0.962 | 40.25 | 60 | | -21 | 27 |
| IC54 | Superior frontal gyrus | 0.939 | 47.11 | -1 | | 12 | 62 |
| IC56 | Precentral gyrus | 0.929 | 34.29 | -3 | | -21 | 57 |
| IC60 | Precentral gyrus | 0.941 | 36.59 | -18 | | -15 | 69 |
| **Cognitive Control Networks (CCN)** | | | | | | | |
| IC15 | Superior orbitofrontal gyrus | 0.977 | 36.15 | -18 | | 60 | -6 |
| IC27 | Posterior insula | 0.968 | 39.09 | 39 | | -3 | 3 |
| IC28 | Anterior insula | 0.969 | 45.74 | 33 | | 24 | -3 |
| IC31 | Superior orbitofrontal gyrus | 0.973 | 40.42 | -18 | | 33 | -21 |
| IC36 | Superior frontal gyrus | 0.973 | 43.6 | 30 | | 57 | 12 |
| IC37 | ParaHippocampus | 0.971 | 40.79 | 21 | | -3 | -27 |
| IC43 | Middle orbitofrontal gyrus | 0.946 | 42.16 | -36 | | 48 | -3 |
| IC45 | Middle frontal gyrus | 0.937 | 33.18 | 24 | | 27 | 45 |
| IC58 | Middle frontal gyrus | 0.904 | 35.29 | 30 | | 51 | 21 |
| IC61 | Hippocampus | 0.913 | 34.25 | 18 | | -36 | 0 |
| **Default Mode Network (DMN)** | | | | | | | |
| IC50 | Angular gyrus | 0.931 | 45.83 | 48 | | -66 | 33 |
| IC55 | Precuneus | 0.956 | 60.00 | 0 | | -57 | 54 |
| IC62 | Medial prefrontal cortex | 0.914 | 44.05 | -3 | | 54 | 30 |
| IC63 | Angular gyrus | 0.899 | 38.59 | 54 | | -48 | 36 |
| IC66 | Posterior cingulate cortex | 0.892 | 40.73 | 3 | | -33 | 24 |
| IC71 | Precuneus | 0.903 | 52.81 | -9 | | -72 | 36 |
| IC77 | Angular gyrus | 0.853 | 41.45 | -48 | | -66 | 33 |
| IC79 | Anterior cingulate cortex | 0.858 | 46.73 | 3 | | 36 | 15 |
| IC80 | Medial prefrontal cortex | 0.908 | 44.67 | 0 | | 51 | -3 |

**Supplementary 5**

Identified predictive features and weights

See Table S2.

Table S2. The identified predictive features and their corresponding weights

| **The predictive features** | **Weights** |
| --- | --- |
| SOFC-mPFC (IC15-IC62) | 0.431 |
| Baseline SID score | 0.365 |
| Postcentral gyrus-Hippocampus (IC3-IC61) | 0.339 |
| SOFC-Middle orbitofrontal cortex (IC15-IC43) | 0.317 |
| Anterior insula-Anterior cingulate cortex (IC28-IC79) | 0.264 |
| Putamen-Precentral gyrus (IC8-IC6) | 0.251 |
| Caudate-Caudate (IC23-IC33) | 0.244 |
| Posterior insula-Anterior insula (IC27-IC28) | 0.239 |
| Middle orbitofrontal cortex-Hippocampus (IC43-IC61) | 0.232 |
| Putamen-Caudate (IC8-IC33) | 0.213 |
| Precentral gyrus-Precentral gyrus (IC6-IC56) | 0.212 |
| Middle frontal cortex-Angular gyrus (IC45-IC77) | 0.212 |
| Hippocampus-mPFC (IC61-IC62) | 0.202 |
| Caudate-SOFC (IC23-IC15) | 0.195 |
| Caudate-Middle frontal cortex (IC33-IC58) | 0.191 |
| Caudate-Superior frontal cortex (IC33-IC36) | 0.179 |
| Putamen-SOFC (IC1-IC31) | 0.178 |
| Putamen-SOFC (IC1-IC15) | 0.177 |
| Supramarginal gyrus-Middle frontal cortex (IC49-IC28) | 0.172 |
| Hippocampus-mPFC (IC61-IC80) | 0.167 |
| Postcentral gyrus-ParaHippocampus (IC5-IC37) | 0.160 |
| Hippocampus-Angular gyrus (IC61-IC77) | 0.155 |
| Caudate-Angular gyrus (IC33-IC77) | 0.154 |
| Hippocampus-Angular gyrus (IC61-IC50) | 0.134 |
| Caudate-Superior frontal cortex (IC33-IC54) | 0.118 |
| Putamen-Caudate (IC1-IC33) | 0.111 |
| Superior frontal cortex-ParaHippocampus (IC54-IC37) | 0.107 |
| Precuneus-Anterior cingulate cortex (IC55-IC79) | 0.102 |
| Thalamus-Anterior insula (IC87-IC28) | 0.096 |
| SOFC-Anterior cingulate cortex (IC15-IC79) | 0.095 |
| Precentral gyrus-ParaHippocampus (IC21-IC37) | 0.082 |
| Thalamus-Middle frontal cortex (IC69-IC45) | 0.078 |
| Precentral gyrus-ParaHippocampus (IC6-IC37) | 0.068 |
| Superior frontal cortex-mPFC (IC36-IC62) | 0.051 |
| Superior frontal cortex-Anterior insula (IC54-IC28) | 0.050 |
| Caudate-Anterior insula (IC33-IC28) | 0.049 |
| SOFC-Precuneus (IC15-IC71) | 0.048 |
| Precentral gyrus-Caudate (IC6-IC33) | 0.016 |
| Duration of disease | 0.005 |

**Abbreviation:** SOFC: Superior orbitofrontal cortex, mPFC: Medial prefrontal cortex, IC: Independent component, SID: Symptom Index of Dyspepsia.

**Supplementary 6**

Between-group differences in predictive features

See Table S3.

Table S3. the between-group differences in these 39 predictive features

| **No.** | **The predictive features** | **Responders** | **Non-responders** | ***t* value** | ***p* value** |
| --- | --- | --- | --- | --- | --- |
| rsFC01 | Caudate-Putamen (IC33-IC1) | -0.06±0.16 | -0.17±0.19 | 3.071 | 0.003* |
| rsFC02 | Caudate-Putamen (IC33-IC8) | -0.02±0.15 | -0.09±0.14 | 2.680 | 0.009* |
| rsFC03 | Caudate-Caudate (IC33-IC23) | 0.02±0.19 | -0.11±0.22 | 3.058 | 0.003* |
| rsFC04 | Precentral gyrus-Putamen (IC6-IC8) | 0.28±0.21 | 0.16±0.21 | 2.622 | 0.010* |
| rsFC05 | Precentral gyrus-Caudate (IC6-IC33) | 0.03±0.17 | -0.07±0.17 | 2.485 | 0.015* |
| rsFC06 | Superior frontal gyrus-Caudate (IC54-IC33) | 0.08±0.18 | -0.06±0.19 | 3.583 | 0.001* |
| rsFC07 | Precentral gyrus-Precentral gyrus (IC56-IC6) | 0.27±0.22 | 0.13±0.20 | 2.974 | 0.004* |
| rsFC08 | SOFG-Putamen (IC15-IC1) | -0.07±0.17 | -0.18±0.13 | 3.417 | 0.001* |
| rsFC09 | SOFG-Caudate (IC15-IC23) | -0.02±0.17 | -0.11±0.17 | 2.760 | 0.007* |
| rsFC10 | Anterior insula-Caudate (IC28-IC33) | 0.05±0.16 | -0.06±0.17 | 3.680 | <0.001* |
| rsFC11 | Anterior insula-Thalamus (IC28-IC87) | 0.25±0.18 | 0.14±0.17 | 3.309 | 0.001* |
| rsFC12 | Anterior insula-Supramarginal gyrus (IC28-IC49) | 0.33±0.17 | 0.20±0.17 | 3.437 | 0.001* |
| rsFC13 | Anterior insula-Superior frontal gyrus (IC28-IC54) | 0.51±0.19 | 0.40±0.19 | 2.865 | 0.005* |
| rsFC14 | Anterior insula-Posterior insula (IC28-IC27) | 0.39±0.15 | 0.30±0.17 | 2.560 | 0.012* |
| rsFC15 | SOFG-Putamen (IC31-IC1) | -0.04±0.14 | -0.14±0.17 | 3.004 | 0.003* |
| rsFC16 | Superior frontal gyrus-Caudate (IC36-IC33) | 0.08±0.13 | -0.02±0.18 | 2.983 | 0.004* |
| rsFC17 | ParaHippocampus-Postcentral gyrus (IC37-IC5) | 0.07±0.17 | -0.05±0.14 | 3.445 | 0.001* |
| rsFC18 | ParaHippocampus-Precentral gyrus (IC37-IC6) | 0.02±0.20 | -0.11±0.18 | 2.969 | 0.004* |
| rsFC19 | ParaHippocampus-Precentral gyrus (IC37-IC21) | 0.01±0.17 | -0.08±0.15 | 2.483 | 0.015* |
| rsFC20 | ParaHippocampus-Superior frontal gyrus (IC37-IC54) | -0.08±0.17 | -0.20±0.18 | 3.545 | 0.001* |
| rsFC21 | Middle orbitofrontal cortex- SOFG (IC43-IC15) | 0.12±0.21 | 0.01±0.21 | 2.574 | 0.012* |
| rsFC22 | Middle frontal gyrus-Thalamus (IC45-IC69) | 0.02±0.19 | -0.09±0.15 | 2.862 | 0.005* |
| rsFC23 | Middle frontal gyrus-Caudate (IC58-IC33) | 0.07±0.17 | -0.03±0.17 | 2.904 | 0.005* |
| rsFC24 | Hippocampus-Postcentral gyrus (IC61-IC3) | 0.15±0.16 | 0.06±0.19 | 2.679 | 0.009* |
| rsFC25 | Hippocampus-Middle orbitofrontal cortex (IC61-IC43) | 0.03±0.15 | -0.10±0.18 | 3.502 | 0.001* |
| rsFC26 | Angular gyrus-Hippocampus (IC50-IC61) | 0.04±0.19 | -0.10±0.21 | 3.270 | 0.002* |
| rsFC27 | mPFC-SOFG (IC62-IC15) | 0.13±0.21 | -0.01±0.21 | 3.060 | 0.003* |
| rsFC28 | mPFC-Superior frontal gyrus (IC62-IC36) | 0.20±0.18 | 0.08±0.23 | 2.469 | 0.016* |
| rsFC29 | mPFC-Hippocampus (IC62-IC61) | -0.08±0.17 | -0.20±0.20 | 3.115 | 0.002* |
| rsFC30 | Precuneus-SOFG (IC71-IC15) | 0.26±0.19 | 0.16±0.17 | 2.749 | 0.007* |
| rsFC31 | Angular gyrus-Caudate (IC77-IC33) | -0.09±0.19 | -0.02±0.14 | -1.821 | 0.072 |
| rsFC32 | Angular gyrus-Middle frontal gyrus (IC77-IC45) | 0.40±0.21 | 0.30±0.15 | 2.383 | 0.019* |
| rsFC33 | Angular gyrus-Hippocampus (IC77-IC61) | 0.07±0.18 | -0.09±0.18 | 4.347 | <0.001* |
| rsFC34 | Anterior cingulate cortex-SOFG (IC79-IC15) | -0.01±0.15 | -0.14±0.17 | 3.871 | <0.001* |
| rsFC35 | Anterior cingulate cortex-Anterior insula (IC79-IC28) | 0.40±0.19 | 0.30±0.17 | 2.722 | 0.008* |
| rsFC36 | Anterior cingulate cortex-Precuneus (IC79-IC55) | 0.22±0.19 | 0.10±0.15 | 2.896 | 0.005* |
| rsFC37 | mPFC-Hippocampus (IC80-IC61) | 0.11±0.18 | 0.01±0.17 | 2.644 | 0.010* |
| Clinical01 | Baseline SID score | 4.49±1.33 | 2.76±0.94 | 6.611 | <0.001* |
| Clinical02 | Duration of disease | 33.63±24.51 | 54.39±39.37 | -2.876 | 0.005* |

**Abbreviation:** SOFC: Superior orbitofrontal cortex, mPFC: Medial prefrontal cortex, IC: Independent component, SID: Symptom Index of Dyspepsia. * *p _FDR_* < 0.05
